# A rare case of the simultaneous location of Echinococcus multilocularis in the liver and the head of the pancreas: case report analysis and review of literature

**DOI:** 10.1186/s12879-019-4274-y

**Published:** 2019-07-24

**Authors:** Marek Kowalczyk, Waldemar Kurpiewski, Ewa Zieliński, Dariusz Zadrożny, Łukasz Klepacki, Wit Juśkiewicz, Jacek Lasocki, Łukasz Dyśko, Krzysztof Batia, Wiesław Pesta

**Affiliations:** 1 Department of Laboratory Medicine Faculty of Health Sciences, University of Warmia and Mazury in Olsztyn, Olsztyn, Poland; 2 Clinic of Oncological and General Surgery, University Clinical Hospital in Olsztyn, Olsztyn, Poland; 3 Department of Emergency Medicine and Disaster Collegium Medicum in Bydgoszcz, Nicolaus Copernicus University, Toruń, Poland

**Keywords:** Echinococcus multilocularis, Hydatis dsease, Pancreas

## Abstract

**Background:**

Echinococcosis multilocularis (Hydatid Disease - HD) is a zoonotic disease caused by the larval form of Echinococcus multilocularis (EM). The main sites for this zoonosis are the Middle East, China, India, Alaska, and Siberia. It is rather rare in Europe. In Poland, the Warmian-Masurian Province is the endemic region for Echinococcus multilocularis. The clinical manifestation of the disease is dependent on the location, the size of the cyst and the development stage of the parasite. Considering the uncommon character of echinococcosis in Central Europe, especially such located in the areas outside the liver and lungs, the authors would like to present a case of coexistence in one patient of two EM foci in the liver and the head of the pancreas.

**Case presentation:**

We present a clinical case of a 32-year-old man who was diagnosed with a cystic lesion with septa and calcification in the sixth segment of the liver and a suspicious change in the head of the pancreas. ELISA Em 2 plus test was positive, Western Blot method - the P-5 pattern showed an image that is characteristic of an EM infection. The sixth liver segment with a tumour and a tumour from the head of pancreas were excised by means of laparotomy. On the 6th day after the surgery the patient was discharged from hospital without complications and in good condition. Currently, he is under the control of a parasitic and zoonotic clinic. He takes an 800 mg daily dosage of Albendazole.

**Conclusions:**

The presented clinical case shows that if we have a patient with cystic / tumour change in the pancreas and positive immunological tests, CT and MRI of the abdominal cavity are usually sufficient in order to fully diagnose and to qualify such a person for surgery. The most effective treatment is surgical treatment supplemented with pre- and postsurgical treatment with Albendazole.

## Background

Echinococcosis (hydatid disease) is a zoonotic disease caused by the larvae Echinococcus multilocularis (EM). The infection usually occurs after accidentally ingesting the parasite’s eggs. The oncospheres which are released from the eggs migrate to the internal organs, mainly the liver (50–77%) and lungs (15–47%), where they develop larval forms that form structures composed of small cysts with a diameter of 0.5 to a few millimetres, containing thousands of protoscolices – hydatid cysts [1]. The main geographical sites where this zoonosis occurs are the Middle East, China, India, Alaska, and Siberia [1, 2]. It is rather rare in Europe. In Poland, the Warmian-Masurian Province is the endemic region for Echinococcus multilocularis. The results from 2002 to 2006 studies show that in this area the occurrence of Echinococcus hosts is the highest - 39.6% among foxes and 5% among raccoon dogs [3]. The rabies eradication carried out throughout Poland since 2002 had the greatest impact on the increase in the number of infected foxes had.

The clinical manifestation of the disease depends on the location, size of the cyst and the development stage of the parasite. The main symptoms in the abdominal location are abdominal pain, epigastric discomfort, vomiting and gradual weight loss. Diagnosis is based on imaging studies, mainly ultrasonography and computed tomography of the abdominal cavity. However, the diagnosis is quite difficult because changes shown in imaging often resemble neoplastic tumours. The enzyme-linked immunosorbent assays (ELISA, Western Blot) are very helpful in differentiating between them. However, laparotomy or laparoscopy becomes the final diagnostic and therapeutic method due to the chance of obtaining false-positive or false-negative results of these tests. Surgical procedures are the most effective treatment methods [4].

Taking into account the rare nature of echinococcosis in Central Europe, especially such affecting areas outside the liver and lungs, the authors would like to present a case of coexistence in one patient of two Echinococcus multilocularis foci in the liver and the head of the pancreas.

## Case presentation

A 32-year-old man reported to the general practitioner with general weakness, weight loss (12% in 6 months), and worsening stomach aches for about a month. The patient has not been treated for chronic diseases so far, he has not been taking any medications permanently. He lives in a city agglomeration. There are no animals in the house. After carefully collecting the family history, including the history of his childhood, it turned out that as a child he went to the forest very often and consumed unwashed forest fruits. The patient told, that he had eaten lots of unwashed berries and blueberries in the forest, immediately after picking them up about 10–15 years before the diagnosis. Abdominal cavity ultrasound revealed a cystic lesion with a septum and calcification in the sixth segment of the liver and a suspected solid change in the head of the pancreas.

In the physical examination no deviations from the normal state were found. In laboratory tests, the following deviations from the normal state were observed: Alat-61 U / i (ref. value < 41 U / l), IgE -182,1 IU / ml (ref. value < 100 IU / ml) CA - 19.9 – normal, CEA – normal.

**Fig. 1:**
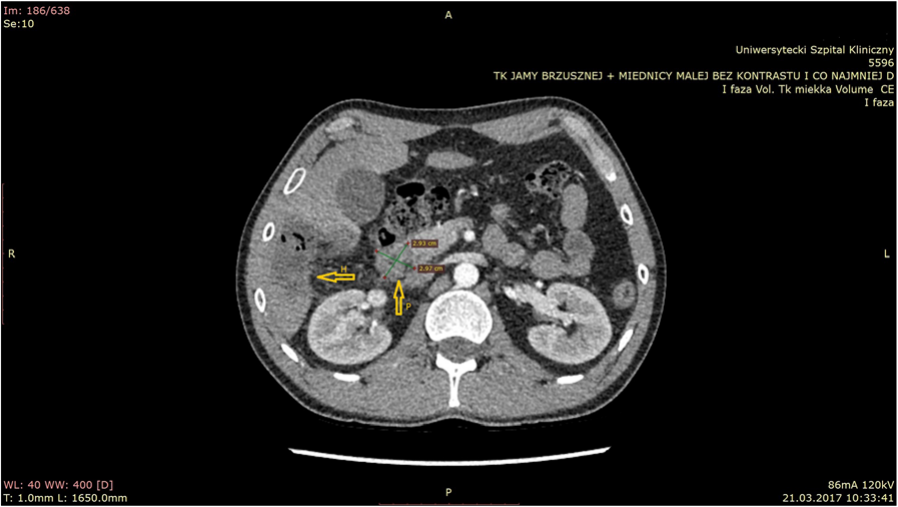
By arrows, a bilelic cyst in the sixth segment of the liver (H) and the head of the pancreas (P) was marked

The CT scans showed that chest organs were normal. The scan of the chest, abdominal and pelvic regions was performed after intravenous contrast administration with a slice thickness of 5 mm. In the abdominal cavity in the 6th liver segment a cystic change was found with calcification in the centre and small septum with dimensions of 43x28x43mm and a solid change with calcified septa with dimensions of 35x29mm in the head of the pancreas, which models the duodenum and the wall of the inferior vena cava (Fig. 1).

**Fig. 2:**
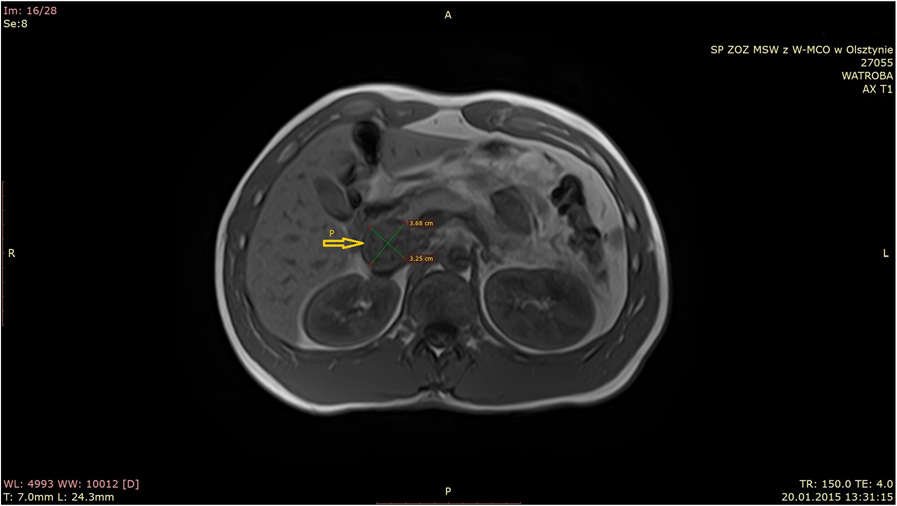
The arrow is used to mark the pancreatic cyst of the pancreas head (P)

The diagnosis embraced also an MR of the abdominal cavity, in which the presence of a change in the liver was confirmed. It showed a weaker signal in T1 images and a heterogeneous T2 signal. The change did not enhance after contrast administration and did not show signs of diffusion limitation. In addition, there was a change of 44x31x42 mm in the head of the pancreas, which models the duodenum and the inferior vena cava (Fig. 2).

A suspicion of echinococcosis was raised and the patient was referred to a specialist dealing with parasitic diseases. ELISA Em 2 plus test was positive, Western Blot method - the P-5 pattern showed an image that is characteristic of an EM. According to the WHO-IWGE PNM [5] recommendations, the disease was classified as P1N1M0.

Based on the exposures, imaging examinations, and tests: ELISA Em 2 plus and Western blot, Echinococcosis multilocularis was diagnosed. Albendazole 400 mg treatment was started in two daily doses, with the control of the concentration of the drug in the blood serum. After 2 years of treatment with Albendazole, the patient was referred to our centre to be qualified for surgery. After analysing the case it turned out that tumour markers (CA.19–9, CEA) were within the normal range, family history of gastrointestinal tumours were negative; imaging tests were carried out: ELISA test, Western blot - confirmation of EM infection. Therefore, we decided to remove the pancreatic and liver changes at the same time.

The sixth liver segment with a tumour and a tumour from the head of pancreas were excised by means of laparotomy.

The macroscopic analysis showed that the liver tumour was in the shape of an irregular cone with a height of 65 mm, after cross-section it consisted of yellow, irregular masses and small cysts filled with a jellylike content.

The tumour of the pancreas head after the excision was 43x30x45 mm and appeared to be surrounded by a thin bag. The microcysts present in cross section were filled with a jellylike content.

**Fig. 3:**
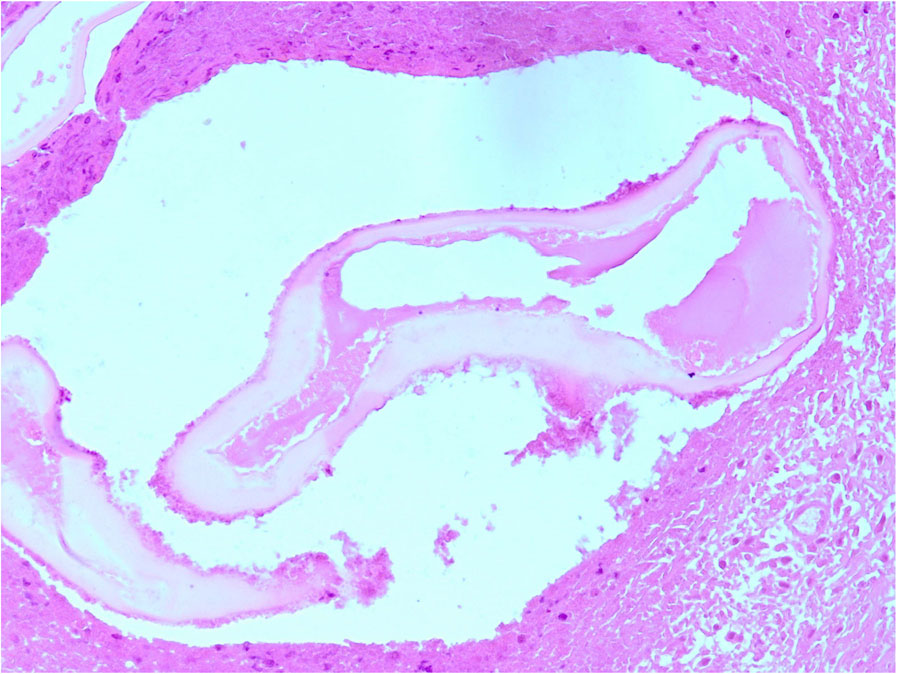
EM-The characteristic laminated cyst wall.(H&E stain)

Histopathological examination confirmed the presence of EM in the liver and in the pancreas (Fig. 3).

In the PCR study, the genetic material of EM was confirmed. Postsurgical course without complications. On the 6th day after surgery the patient was discharged from hospital without complications and in good condition.

**Fig. 4:**
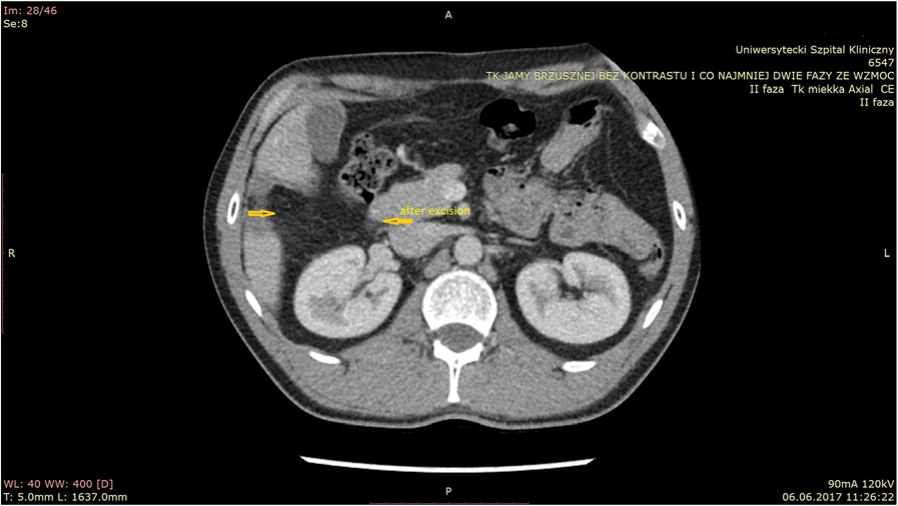
The arrows marked the place after cutting out both cysts

Currently, he is under the control of a parasitic and zoonotic clinic. He takes an 800 mg daily dosage of Albendazole. The follow-up CT of the abdomen performed 2 months after the surgery showed no recurrence in the abdominal cavity (Fig. 4).

## Discussion and conclusion

Hydatid disease is most commonly located in the right lobe of the liver. Pancreatic localisation is described in the literature with a frequency of 0.2–2% [6] Most often, lesions are located in the head of the pancreas (50–58%). Location in the body and tail of the pancreas is found in 24–34 and 19%, respectively [7].

In our case, the changes in the liver and pancreas were similar in size and probably developed independently at the same time. There are several paths of the development of the EM in the body of a human host. Larvae with a diameter of 0.3 mm and smaller that have escaped the hepatic filtering system (first Lemman’s filter) can migrate to the lungs, which are the most frequent place where EM is located after the liver. If the larvae are not stopped in the lungs (second Lemman’s filter), they can into the bloodstream and to other organs.

The oncospheres released in the small intestine penetrate through the mucous membrane into the blood vessels and enter the liver with blood. There is also the possibility of them getting into the lymphatic vessels. This migration of EM larvae resembles the spread of digestive tract tumours.

Due to their small size, EM larvae can get back to the pancreas from the liver through the bile duct [1, 8, 9]. Another way available for larvae leads through a well-developed lymphatic vessels network in the pancreatic and hepatic region. It is also possible for larvae to migrate back through a well-developed network of venous vessels that drain the blood from the pancreas to the liver. This route seems especially likely in people with portal hypertension or obstruction of blood outflow from the vessels supplying the portal vein. The portal venous system is not completely closed. It combines multiple anastomoses with the system of main veins in many places and this way EM larvae have also a possibility to enter. Tumour cells from the pancreas can migrate taking as similar path as the EM larvae. Therefore, the location of EM pancreatic changes and metastatic changes of pancreatic adenocarcinoma to the liver may be comparable [10] Some sources provide the possibility of spreading EM larvae through loose connective tissue taking the retroperitoneal route [11].

Data from various sources describe hydatid disease in the form of a cyst. Solid changes are described sporadically. The clinical manifestation of the disease depends on the location, size of the cyst and the development stage of the parasite. Pancreatic cysts in the human pancreas grow slowly (3-20 mm / year), which is why clinical symptoms may appear late, especially if the change grows outside the organ and does not pressure the relevant clinical and anatomical structures (bile duct, pancreatic duct, splenic vein, duodenum) [12]. Cystic / tumour symptoms depend on the location of the lesion in the pancreas. Most often, these are upper abdominal pain with a palpable or imperceptible tumour and accompanying jaundice. It is also accompanied by a feeling of epigastric discomfort, nausea, vomiting, and gradual weight loss. Jaundice most often results from the pressure on the bile ducts or fistulas of the cyst to the main bile duct with associated symptoms of cholangitis [13]. This applies mainly to changes in the head of the pancreas. Cysts located in the distal part of the body and tail of the pancreas later give clinical symptoms and often cause compression of the adjoining organs, which is manifested by the obstruction of the upper gastrointestinal tract, splenic vein thrombosis with regional portal hypertension. Spontaneous perforation of the cyst into the free peritoneal cavity was described in 9.3% of cases of echinococcal cyst located near the pancreas [14].

Sometimes the cyst undergoes secondary superinfection. This occurs in the form of an abscess with acute abdominal symptoms. The literature also describes fistulas of the alimentary tract [13]. Complications are diagnosed during presurgical diagnostics or only during surgery.

Diagnosis of pancreatic cyst / tumour is based on the use of all of the available imaging tests - mainly USG, CT and abdominal MR. However, it is quite difficult because changes shown in imaging studies often resemble benign and malignant changes observed in the pancreas. Different types of EM antigens are very helpful in differentiating. Patients with positive immunological tests have a more frequent location of the cyst in the liver than other organs, although seronegativity does not exclude the extrahepatic location [15].

It should be remembered that in patients with pancreatic EM localisation, bilirubin, aminotransferases, alkaline phosphatase, lipase, Ca-19.9 and CEA should be determined. Casoni’s test, which is still being carried out in some centres, has low sensitivity and is often false negative. We rely on ELISA and Western Blot test. However, due to the possibility of obtaining false-positive or false-negative results of these tests, often the final diagnostic and therapeutic method becomes a laparotomy or laparoscopy, which in combination with histopathological examination of the acquired tissue material gives 100% certainty of the diagnosis. Some authors report that 40–49% of EM invasions in the pancreatic localisation [5] can be confirmed before the surgery. If we use a wider panel of EM: Em2, Em2+, Em18 antigens for presurgical diagnostics, the sensitivity increases to 90–100% and the specificity to 95–100% [5, 16–19].

Detection of EM DNA by means of PCR is also an accessible and recognised method, but the reservoir material is required before surgery. Fearful of echinococcosis and symptoms of anaphylaxis, some authors do not recommend an aspiration biopsy, although PAIR is one of the techniques for the treatment of hydatid cyst which is generally recognised [13].

A patient with cystic / tumour change in the pancreas and positive immunological tests, CT and MRI abdominal cavity, which are generally available and non-invasive, are usually sufficient to obtain a full diagnosis and qualify the patient for surgery [20].

In case of complicated cysts, invasive tests like EUS and ERCP must be used for the diagnosis [15].

When during the surgery we are not sure if there is a connection between the cysts with the bile, pancreatic or digestive tract, one can use the contrast administration to the cyst or perform intrasurgical cholangiography. You can use ERCP to perform intrasurgical bile duct prosthesis [15].

The most effective treatment is surgery. Percutaneous drainage of pancreatic echinococcal cyst is an alternative to surgical treatment in people with high intrasurgical risk [15]. Surgical treatment can be radical or sparing. Radical procedures are associated with a lower risk of relapse, but they carry a greater risk of postsurgical complications. Postsurgical recurrence’s frequency is around 2–5% and is most often caused by incomplete cyst excision, leaving undiagnosed cysts and due to the lack of protection from Albendazole [21].

Presurgical use of Albendazole drugs reduces the risk of dissemination, anaphylaxis and recurrence of echinococcosis in patients operated on or treated with the use of the PAIR procedure. They are used at least 1 month before surgery or PAIR, while the length of application after surgery depends on the treatment centre. A minimum 2-year treatment period with Albendazole is recommended [22]. It should be remembered that albendazole has a parasitostatic effect. It is not a parasiticidic drug and its discontinuation may cause the disease to come back. This should be remembered especially after liver transplantation in patients with echinococcosis multilocularis. An additional factor in the recurrence of the disease are immunosuppressive drugs taken by these patients.

Complications of surgical treatment can be early or late. Perioperative mortality ranges from 0.5 to 4.0% [23]. The mortality rate of untreated patients exceeds 90% in 10 years [3]. The most frequent complications of the surgical treatment of echinococcosis multilocularis include pancreatic fistula, biliary fistula, intra-abdominal abscess, and postsurgical wound infection. In the case of biliary or pancreatic fistula, an MRCP and ERCP should be performed, which allows simultaneous prosthesis of the bile and pancreatic ducts as one of the methods of treatment. In the literature you can find descriptions of pancreatic fistula treatment with somatostatin analogs [24].

In the case of intra-abdominal abscess and postsurgical wound infection, you should follow a routine procedure.

The main late complications are recurrent cysts, which are rather rare in case of radical surgical treatment with the use of Albendazole [25]. In the postsurgical course, Albendazole should be administered for at least 2 years, and in many recurrent or inoperable cases, the complications and intolerance of treatment with Albendazole should be taken into consideration, which is not a frequent phenomenon, fortunately.

Based on exposure, clinical picture, imaging tests, and serological tests results Echinococcosis multilocularis can be diagnosed with sensitivity of up to 90–100% and specificity of up to 95–100%. The pancreatic EM location is a rare form of HD. The most effective treatment is radical surgical treatment combined with Albendazole treatment.

## Data Availability

Please contact author for data requests: Marek Kowalczyk e-mail: forel@neostrada.pl

## Abbreviations

Alat: Alanine transaminase
CA 19–9: Carbohydrate antigen 19–9
CEA: Carcinoembryonic antigen
CT: Computed Tomography
ERCP: Endoscopic Retrograde Cholangiopancreatography
EUS: Endoscopic Ultrasound
IgE: Immunoglobulin (Ig) “isotype” E
MR: magnetic resonance
MRI: Magnetic Resonance Imaging
PAIR: Puncture-Aspiration-Injection-Reaspiration
PCR: Polymerase chain reaction
USG: Ultrasonography
WHO-IWGE PNM: The World Health Organization-Informal Working Group on Echinococcosis: Parasitic mass in the liver (P), the involvement of neighbouring organs (N), and metastases (M)
